# Diminished ovarian reserve is associated to euploidy rate: a single center study

**DOI:** 10.3389/fendo.2024.1535776

**Published:** 2025-01-17

**Authors:** Edoardo Carnesi, Stefano Castellano, Elena Albani, Andrea Busnelli, Antonella Smeraldi, Ozgur Bulbul, Emanuela Morenghi, Valentina Immediata, Paolo Emanuele Levi-Setti

**Affiliations:** ^1^ Division of Gynecology and Reproductive Medicine, Department of Gynecology, Fertility Center, Istituto di Ricerca e Cura a Carattere Scientifico (IRCCS) Humanitas Research Hospital, Milan, Italy; ^2^ Genomic Medicine Research Center, Department of Medicine and Surgery, University of Insubria, Varese, Italy; ^3^ Department of Biomedical Sciences, Humanitas University, Milan, Italy

**Keywords:** AMH, assisted reproduction (ART), PGT-A, diminished ovarian reserve (DOR), embryo ploidy rate

## Abstract

**Background:**

Reproductive success shows a well-documented decline with advancing maternal age, primarily due to chromosomal abnormalities (aneuploidies) in embryos. While ovarian reserve markers such as Anti-Müllerian Hormone (AMH) and Antral Follicle Count (AFC) traditionally serve as quantitative predictors of fertility, emerging evidence suggests they may also reflect oocyte quality, particularly in patients with Diminished Ovarian Reserve (DOR). The relationship between these biomarkers and embryo chromosomal status remains complex and poorly understood.

**Methods:**

We conducted a retrospective analysis of *in vitro* fertilization (IVF) cycles performed between 2015 and 2022, involving 773 female patients who underwent IVF and pre-implantation genetic screening for aneuploidy (PGT-A). Our patient cohort was divided into two groups: Group 1, consisting of women who achieved at least one euploid embryo, and Group 2, comprising women who did not.

**Results:**

The main outcome measures included the rate and number of euploid blastocysts and their correlation with ovarian reserve. Our results showed a statistically significant association between independent variables and embryo ploidy: AMH levels (OR 1.09; 95% CI 1.04-1.14, p<0.001), the age of the woman (OR 0.82; 95% CI 0.79-0.85, p<0.001), the number of oocytes retrieved (OR 1.050; 95% CI 1.01-1.08, p=0.05), and the fertilization rate (OR 6.69; 95% CI 2.67-16.77, p<0.001).

**Conclusion:**

Our findings suggest that AMH levels are associated with embryo ploidy rate. These insights could enhance counseling practices in assisted reproductive technology (ART), offering patients a more detailed understanding of their infertility prognosis and the factors influencing IVF outcomes.

## 1 Introduction

It is widely known that there is a strong correlation between female age and the chances of spontaneous conception as well as success with assisted reproductive technologies (ART) (1). This appears to be due to the increased risk of having aneuploid embryos as age increases (2). Another known prognostic factor for ART is ovarian reserve parameters. These indices are important for decision-making, as they provide valuable insights into a woman’s fertility potential. To be effective, ovarian reserve tests (ORTs) need to be user-friendly, reliable, and easily interpretable for follow-up assessments (3).

Anti-Müllerian Hormone (AMH) is a glycoprotein hormone produced by the granulosa cells of the ovarian follicles (4).

AMH signaling is mediated through the AMH type II receptor (AMHRII), which is a serine/threonine kinase receptor (5). The binding of AMH to AMHRII leads to the recruitment and phosphorylation of the AMH type I receptors (ACVR1, BMPR1A, and BMPR1B), which then activate the SMAD signaling pathway (5). This event results in the transcriptional regulation of target genes involved in follicular development and ovarian function (5).

AMH has two key roles in regulating ovarian function: the first is the inhibition of primordial follicle recruitment (5). More in detail, AMH inhibits the initial recruitment of primordial follicles into the growing follicle pool, thereby maintaining the primordial follicle reserve (5). The second role is the modulation of follicular growth, i.e. AMH inhibits the growth of preantral and small antral follicles, slowing their maturation and selection of the dominant follicle (5).

Among the various markers used to assess ovarian reserve, the anti-Mullerian hormone (AMH) and antral follicle count (AFC) stand out as the most predictive indicators of ovarian response to ovarian stimulation. These markers have demonstrated superior reliability in predicting poor ovarian response (POR) compared to other indicators (6–8). Moreover, they are considered highly accurate in forecasting the response to controlled ovarian stimulation, particularly in the context of *in vitro* fertilization (IVF) treatments (9). Despite recent evidences have shown significant associations between age, AFC, and AMH with embryo quality, the relationship between diminished ovarian reserve (DOR) and an increased risk of aneuploid pregnancies is still a subject of mixed evidence (10, 11). Some researchers revealed that DOR did not exhibit an association with elevated occurrences of aneuploidies among younger women undergoing IVF to achieve pregnancy (12, 13). Conversely, others suggested that blastocysts derived from women with DOR were less likely to be euploid compared to those from women without DOR, even after adjusting for age. The observed reduction in euploid rates correlated with the quantity of oocytes retrieved in these studies (14–16).

The same pattern leading to mixed evidence is found when taking into account papers on non-elderly patients. Indeed, several studies have found an association between low AMH levels and increased aneuploidy rates (17). On the contrary, other authors reported that AMH levels did not differ between women with an aneuploid fetus and those with a euploid fetus (18). Additionally, a secondary analysis of the EAGER study found no statistically significant association between low AMH and fecundability in women with a prior pregnancy loss (19).

Other studies lie in the midst of these perspectives, concluding that serum AMH levels and AFC biomarkers may correlate with both quantitative and qualitative aspects of ovarian reserve within DOR patients’ subgroup. However, due to limitations inherent in the studies analyzed, the underlying cause of this effect remains unclear; this way, establishing a causal relationship between DOR and heightened miscarriage rates due to aneuploidies remains unclear (12–16, 20).

In light of this knowledge, we investigated the association between ovarian reserve markers (AMH and AFC) and euploidy rate in couples undergoing ART and preimplantation genetic testing for aneuploidies (PGT-A).

## 2 Materials and methods

### 2.1 Patients inclusion criteria

The present retrospective monocentric study was carried out at Humanitas Research Hospital, Rozzano (Milan, Italy). We evaluated the outcomes of 773 female patients, who underwent both IVF and comprehensive chromosomal screening between 2015 and 2022, selected according to the criteria described below.

We included in the study women with advanced maternal age (AMA), between 35 and 45 years, history of recurrent failure in IVF cycles (RIF) - two or more prior cycles, unexplained recurrent pregnancy loss (RPL) (21).

All women underwent baseline follicular phase ultrasound evaluations for assessing the AFC. Blood samples were collected to measure follicle-stimulating hormone (FSH, on cycle day 2 or 3), and AMH (level greater than 1.2 ng/mL was considered normal) (22). AMH lower than 1.2 ng/mL and AFC lower than 10 have been defined as cut-offs for reduced ovarian reserve.

Severe dyspermic semen samples, according to World Health Organization guidelines, and TeSE samples were excluded from the study. Samples included in the study are expressed as total progressive motile count (TPMC) millions/ejaculate (23).

### 2.2 Clinical procedures

Ovarian stimulation and oocyte retrieval were performed as described in detail Ragni, Cardellicchio, Cirillo, Somigliana and colleagues (24–27). The protocol and dosage for each patient were tailored based on age, weight, prior response to stimulation and the results of ovarian reserve testing, as described in ESHRE guidelines (28).

The traditional Intracytoplasmic Sperm Injection (ICSI) treatments employed fresh ejaculated semen samples with heterogeneous quality. Embryo culture, and blastocyst biopsy procedures were employed according to internal protocols and quality control standards. Trophectoderm biopsy was conducted on every expanding or completely expanded blastocyst on post-retrieval day 5, 6 and 7, depending on the developmental stage reached by each individual embryo (monitored through time-lapse incubators and graded through the guidelines of ESHRE’s Istanbul consensus) (29).

In-depth PGT-A was conducted on each trophectoderm biopsy sample as described by recent literature (30).

### 2.3 Study design and statistical analysis

Our patient cohort was stratified into two groups: Group 1, consisting of women who obtained at least one euploid blastocyst, and Group 2, consisting of women who failed to achieve euploid blastocysts. Within the two groups, we also compared AMH levels of patients by further age stratification (<40, ≥40 years).

Data were expressed as number and proportion, if categorical, or mean and standard deviation (± SD), if continuous.

Differences between the two groups were explored with the chi-square test, for continuous variable, or Mann Whitney test, for continuous variable, due to non-Gaussian distribution.

Associations with euploidy rate were explored with fractional response regression. All independent variables showing a p under 0.2 were then submitted to a multivariable fractional response regression analysis. The results were expressed as Odds Ratio (OR) and 95% confidence interval (95%CI). Euploidy was also considered as a dichotomous variable, with 1 indicating the presence of at least one euploid embryo. The association was then explored with logistic regression analysis. All independent variables showing a p under 0.2 were then submitted to a multivariable logistic regression analysis. The results were expressed as odds ratio (OR) and 95% confidence interval (95%CI). Significance threshold was set to 0.05. All analyses were made with Stata version 18.

## 3 Results

### 3.1 Baseline characteristics

The baseline characteristics of the studied population are summarized as mean values (± SD). A total of 773 couples were included in the analysis. The mean age of the women was 39.9 ± 3.2 years, while the mean age of the male partners was 41.9 ± 5.1 years. The mean female body mass index (BMI) was 21.9 ± 3.1. Ovarian reserve markers showed an average AMH level of 3.03 ± 2.12 ng/mL, FSH 7.62 ± 2.41 IU/L, and an AFC of 13.9 ± 7.8.

In Group 1 (n = 439), women were younger, with a mean age of 38.9 ± 3.3 years, compared to their male partners at 41.1 ± 4.9 years. The mean female BMI was 22.1 ± 3.1, with corresponding AMH, FSH, and AFC values of 3.37 ± 2.37 ng/mL, 7.47 ± 2.29 IU/L, and 14.5 ± 7.9, respectively.

In contrast, Group 2 (n = 334) showed higher mean ages for both women (41.3 ± 2.5 years) and their male partners (43.0 ± 5.2 years). The mean BMI was 21.8 ± 3.2, with AMH, FSH, and AFC values of 2.59 ± 1.64 ng/mL, 7.82 ± 2.57 IU/L, and 13.1 ± 7.7, respectively.

Statistical analysis revealed significant differences between the groups for female and male ages (*P*<0.001), FSH (*P*=0.04), and AFC (*P*=0.007). Baseline characteristics are also reported and compared in **Table 1**.

**Table 1:** Baseline and IVF cycles characteristics of the investigated couples.

	Entire sample	Group 1 (at least 1 eupl. embr.)	Group 2 (no eupl. embr.)	*P* value
n	773	439	334	
Woman age	39.9 ± 3.2	38.9 ± 3.3	41.3 ± 2.5	<0.001
Male age	41.9 ± 5.1	41.1 ± 4.9	43.0 ± 5.2	<0.001
Time of infertility (months)	52.1 ± 31.6	53.8 ± 31.0	49.8 ± 32.4	0.014
BMI	21.9 ± 3.1	22.1 ± 3.1	21.8 ± 3.2	0.092
Active smoke	81 (13.11%)	42 (12.21%)	39 (14.23%)	0.459
AMH	3.03 ± 2.12	3.37 ± 2.37	2.59 ± 1.64	<0.001
FSH	7.62 ± 2.41	7.47 ± 2.29	7.82 ± 2.57	0.040
AFC	13.9 ± 7.8	14.5 ± 7.9	13.1 ± 7.7	0.007
Retrieved oocytes	12.5 ± 5.8	13.5 ± 5.9	11.3 ± 5.4	<0.001
Injected oocytes	8.7 ± 3.4	9.3 ± 3.2	7.9 ± 3.4	<0.001
Fertilization rate	78.1 ± 17.7	80.1 ± 16.1	75.5 ± 19.3	0.002

Results are expressed as mean ± SD.

### 3.2 IVF cycle

IVF cycle characteristics of the entire population examined, expressed as mean (± SD), are as it follows.

Retrieved oocytes: 12.5 (± 5.8); injected oocytes: 8.7 (± 3.4); fertilization rate: 78.1%(± 17.7); fertilized oocytes 6.7(± 3.0). Cleavage embryos: 5097. Biopsied blastocysts: 2,506. Blastulation rate: 48%.

In Group 1, IVF cycle characteristics, expressed as mean (± SD), are as it follows.

Retrieved oocytes: 13.5 (± 5.9); injected oocytes: 9.3 (± 3.2); fertilization rate: 80.1% (± 16.1); fertilized oocytes 7.4(± 2.9). Cleavage embryos: Cleavage embryos: 7.4 (± 2.9). Total number of cleavage embryos: 3160 (62%). Biopsied blastocysts: 1760 (70%). Euploid blastocysts 833/1760 (47%).

In Group 2, IVF cycle characteristics, expressed as mean (± SD), are as it follows.

Retrieved oocytes: 11.3 (± 5.4); injected oocytes: 7.9 (± 3.4); fertilization rate: 75.5% (± 19.3); fertilized oocytes 7 (± 5.6). Cleavage embryos: 6.0 (± 4.2). Total number of cleavage embryos: 1937 (38%). Biopsied blastocysts: 756 (30%). Aneuploid blastocysts: 729. 27 missing blastocysts resulted in inadequate PGT-A analysis, and for this reason were discarded from statistics.

In our analysis we found statistical differences between the two groups according to retrieved oocytes (*P*<0.001), injected oocytes (*P*<0.001) and fertilized oocytes (*P*=0.002).

IVF cycle characteristics are also reported and compared in **Table 1**.

### 3.3 Fractional and logistic regression

According to the fractional regression, in univariable analysis: AMH (OR 1.09; 95%CI 1.04-1.14, *P*<0.001) and woman age (OR 0.82; 95%CI 0.79-0.85, *P*<0.001) exhibited a strong statistically significant association; the number of retrieved oocyte (OR 1.02; 95%CI 1.00-1.04, *P*=0.035) and months of unprotected intercourse (OR 1.003; 95%CI 1.00-1.01, *P*=0.025) showed a significant association. In multivariable analysis, only AMH (OR 1.05, 95%CI: 1.00-1.10, *P*=0.030) and woman age (OR 0.82, 95%CI 0.79-0.85, *P*<0.001) remained significantly associated. **Table 2** reported and summarized all the parameters investigated.

**Table 2:** Logistic regression analysis (univariable and multivariable analysis) of both baseline characteristics and retrieved, injected and fertilization rate.

	Univariable		multivariable	
	OR (95%CI)	p	OR (95%CI)	p
AMH	1.22 (1.13-1.32)	<0.001	1.13 (1.03-1.24)	0.009
FSH	0.94 (0.89-1.00)	0.045		
AFC	1.02 (1.00-1.04)	0.014		
Oocyte retrieved	1.08 (1.05-1.11)	<0.001	1.050 (1.01-1.08)	0.005
Woman age	0.73 (0.68-0.78)	<0.001	0.73 (0.68-0.78)	<0.001
BMI	1.03 (0.98-1.08)	0.233		
Smoking	0.84 (0.52-1.34)	0.459		
Non protected sexual intercourse (Months)	1.00 (1.00-1.01)	0.080	–	
Oocyte injected	1.14 (1.09-1.19)	<0.001		
Fertilization rate	4.45 (1.97-10.09)	<0.001	6.69 (2.67-16.77)	<0.001

Results are expressed as mean ± SD.

According to the logistic regression, in the univariable model, women’s age, AMH, FSH, AFC, retrieved oocytes, injected oocytes, and fertilization rate showed a significant positive association with embryo ploidy. The results are as follows. Women’s age: OR 0.73 (95% CI: 0.68-0.78), *P*<0.001; AMH: odds ratio (OR) 1.22 (95% CI: 1.13-1.32), *P*< 0.001; FSH: OR 0.94 (95% CI: 0.89-1.00), *P*=0.045; AFC: OR 1.02 (95% CI: 1.00-1.04), *P*=0.014; retrieved oocytes: OR 1.08 (95% CI: 1.05-1.11), *P*<0.001; injected oocytes: OR 1.14 (95% CI: 1.09-1.19), *P*<0.001; fertilization rate: OR 4.45 (95% CI: 1.97-10.09), *P*<0.001.

The multivariable logistic regression model incorporated significant predictors identified in the univariable analysis to adjust for potential confounders. The adjusted analysis confirmed that AMH, the number of retrieved oocytes and fertilization rate remain a significant positive predictor of the dependent variable. The results are as follows. AMH: adjusted OR 1.13 (95% CI: 1.03-1.24), *P*=0.009; retrieved oocytes: adjusted OR 1.05 (95% CI: 1.01-1.08), *P*=0.005; fertilization rate: adjusted OR 6.69 (95% CI: 2.67-16.77), *P*<0.001.

In this analysis women’s age maintained a significant negative association: adjusted OR 0.73 (95% CI: 0.68-0.78), *P*<0.001.

The results of the univariable and multivariable logistic regression analysis are summarized in **Table 2**.

### 3.4 AMH and age

Regarding age stratification, the results, expressed as mean (± SD), are as follows.

In Group 1 we found: 230 (52%) ≥40 years women; 209 (48%) <40 years old women; AMH of older women: 3.29(± 2.3); AMH of younger women: 3.43(± 2.3).

In Group 2 we found: 275 (82%) women ≥40 years old; 59 (18%) women <40 years old; AMH of older women: 2.54(± 1.5); AMH of younger women: 2.77 (± 1.6).

Mann-Whitney test evidenced a significant difference between AMH levels belonging to patients of the same age stratification, ≥40 years (*P*<0.0001). Comparing AMH levels of Group 1 older women (≥40 years) with Group 2 younger women (<40 years) we observed a statistical difference (*P*=0.0424).

The results of the age stratification analysis are also reported and compared in **Table 3**.

**Table 3:** Age stratification analysis, according to AMH levels.

	AMH (mean ± SD) Group 1 (at least 1 eupl. embr.)	AMH (mean ± SD) Group 2 (no eupl. embr.)	*P* value
≥40 years old	n=230 (3.29 ± 2.3)	n=275 (2.54 ± 1.5)	p<0.0001
<40 years old	n=209 (3.43 ± 2.3)	n=59 (2.77 ± 1.6)	p=0.8169

Results are expressed as mean ± SD.

## 4 Discussion

The present retrospective study aimed to provide evidences from a large patient’s court on the relationship between DOR and embryo euploidy rate in patients undergoing PGT-A. Our results showed a statistically significant association between woman age, AMH and embryo ploidy.

DOR is influenced by various factors, including autoimmunity and environmental one, indeed oxidative stress, hypoxia, and vitamin D deficiency have been implicated in the pathogenesis of DOR (31–34). Moreover lifestyle factors like smoking, diseases such as endometriosis, and iatrogenic factors like chemotherapy or ovarian surgery can both accelerate follicular depletion and affect ovarian reserve (35–38).

Genetic determinants responsible for DOR remain largely unknown, with only a few genes (GDF9, FMR1, BMP15, and NR5A1) identified as contributors (39–41). This complex array of factors presents significant challenges in developing an effective model for studying DOR.

Age is a well-established factor that negatively impacts ovarian reserve (42). This age-related decline is a natural process and is well-documented in the literature (42–44).

The average age at which women give birth to their first child has increased significantly over the last few decades due to societal changes, including postponing marriage, using contraception, and the introduction of assisted reproductive technology (ART) (45, 46). Biological age is defined by physiology, but it was reported that reproductive aging differed amongst individuals, despite chronological age is a highly significant prognostic factor for fertility and ovarian response in ART cycles (47).

An indicator of an ovary’s biological age appears to be ovarian reserve parameters (47, 48). The quantity and quality of a woman’s eggs, which are essential for fertility, gradually decrease as she moves through her reproductive years. This process shows how responsive the ovarian reserve is to ovarian stimulation (47, 48).

The reduction of ovarian reserve can also have iatrogenic causes, such as chemotherapy. Numerous studies have shown that chemotherapy, particularly regimens containing alkylating agents, can cause direct damage to the primordial follicles, leading to a rapid and irreversible loss of the ovarian reserve (49–54). The degree of ovarian damage depends on the type, dose, and duration of the chemotherapeutic agents used (53, 54). Chemotherapy-induced ovarian damage can result in amenorrhea, premature ovarian failure, and infertility (49–54).

Finally, ovarian surgery, such as cystectomy or oophorectomy, can also have a detrimental impact on ovarian reserve. The removal of ovarian tissue can lead to a direct loss of follicles, resulting in a decreased ovarian reserve (55). Additionally, ovarian and tubal surgery can disrupt the ovarian blood supply, further compromising the remaining ovarian function (56).

The impact of ovarian reserve on oocytes and embryo quality is a widely debated topic in scientific literature, though there are several studies that led to conflicting conclusions (12–15, 20, 57). This might be because there is no universally accepted definition of DOR currently (14, 22, 58).

Despite studies examining DOR patients who underwent IVF as a uniform group produced contrasting results, the relationship between oocytes retrieved and their quality is not well established (59, 60). Some studies have found a link between premature low ovarian reserve and aneuploidy to varying degrees, suggesting a biological link between chromosome segregation and follicular depletion (61, 62).

Studies supporting these findings examined correlations of various attributes, such as oocyte and embryo morphology, rates of aneuploidy in PGT-A, and miscarriage rate (22, 63, 64).

Our findings align with prior research reported in several publications, including those by Katz-Jaffe et al., La Marca et al. and Jaswa et al. (14–16). Our investigation reveals that AMH exhibits a significant association with both embryo ploidy and woman age (*P*<0.001 for both woman age and AMH, as reported in **Tables 2**, **4**). Surprisingly, our data do not demonstrate a direct association of FSH and AFC with embryo ploidy (*P*=0.095 and *P*=0.547, respectively, as reported in **Table 4**). However, when considering patients of Group 1 compared to those of Group 2, significant associations emerge (*P*=0.045 for FSH and *P*=0.014 for AFC, as reported in **Table 2**). These findings indicate no association between BMI or smoking behavior and embryo ploidy (*P*=0.078 and *P*=0.513, respectively, as reported in **Table 4**) and are consistent with those reported by Goldman and colleagues (65). However, when analyzing the differences between women with at least one euploid embryo versus those without, retrieved oocytes and fertilization rate demonstrate significance (*P*=0.05 and *P*<0.001 respectively, as reported in **Table 2**).

**Table 4:** Fractional regression analysis (univariable and multivariable analysis) of both baseline characteristics and retrieved, injected and fertilization rate.

	Univariabile		Multivariable	
	OR (95%CI)	p	OR (95%CI)	p
AMH	1.09 (1.04-1.14)	<0.001	1.05 (1.00-1.10)	0.030
FSH	0.96 (0.91-1.01)	0.095		
AFC	1.00 (0.99-1.02)	0.547		
Oocyte retrieved	1.02 (1.00-1.04)	0.035	–	
Woman age	0.82 (0.79-0.85)	<0.001	0.82 (0.79-0.85)	<0.001
BMI	1.03 (1.00-1.07)	0.078	–	
Smoke	1.14 (0.77-1.70)	0.513		
Unprotected intercourse (Months)	1.003 (1.000-1.007)	0.025	–	
Oocyte injected	1.02 (0.99-1.05)	0.173		
Fertilization rate	1.27 (0.65-2.47)	0.487		

Results are expressed as mean ± SD.

Parameters from the univariable analysis, including FSH, AFC, BMI, smoking status, duration of unprotected intercourse, and the number of injected oocytes, were either not included in the multivariable model or did not retain significance after adjustment for other variables. This suggests that their associations with the dependent variable were not robust to adjustment for other predictors.

Studies have shown that AMH levels decline with advancing age (66). This decline in AMH levels as increasing age is indicative of the gradual decrease in the number of antral follicles and the overall decline in ovarian reserve as women get older (66). Interestingly, we observed a statistical significance between the average AMH levels of the older women belonging to Group 1 (3.29 ± 2.3) and the same parameter of the younger females of Group 2 (2.77 ± 1.6) (*P*=0.0424). **Table 3** also shows statistical significance between AMH levels of ≥40 years women in the two groups. Regarding <40 years women, AMH levels were not statistically significant, as reported in **Table 3**. These findings are consistent with those reported by Li and colleagues, who demonstrated that AMH levels were significantly greater in cycles with at least one euploid embryo compared to cycles with no normal embryos, despite the number of embryos biopsied (67). This finding suggests that the presence of euploid embryos is associated with higher AMH levels, indicating a potential relationship between ovarian reserve, AMH levels, and embryo ploidy status. This disparity in euploid embryo acquisition between age groups aligns with the concept that age influences both ovarian reserve and the likelihood of producing euploid embryos. Additionally, literature indicates that in older women, aneuploidy rates can reach close to 90% and higher miscarriage rate too (20, 68).

Our study exhibits congruent datasets and conclusions with those presented by Katz-Jaffe, La Marca and Jaswa, Arnanz and colleagues, particularly regarding the age of patients, which tends to be older compared to the findings reported by Fouks and colleagues, where the conclusions differ from ours (13–16, 69).

## 5 Conclusion

Data about 773 infertile couples undergoing IVF cycle with PGT-A, experiencing a concomitant decline in ovarian reserve, were presented. Though, the quantitative ovarian reserve assessments of the follicular machinery may reflect relative ovarian aging for some women in light of the concurrent decline in euploid rates and lower ovarian reserve reported in this study. Our data showed that AMH and women’s age are associated with embryo ploidy status; on the other hand, FSH or AFC were not associated with AMH.

Achieving a consensus on the clinical definition of ovarian reserve is crucial and conducting multicenter trials is imperative to gain a comprehensive understanding of this concept.

It is essential to acknowledge that our study might be subject to bias due to the internal policy of the center, which restricts access to PGT-A to women with nearly four blastocysts. Consequently, we lack data on individuals who do not meet these criteria, and in some instances, individuals with four blastocysts are not classified as having diminished ovarian reserve. Additionally, the limitations of this study are those typical of the retrospective studies including: potential confounding bias, generalizability concerns and lack of standardized outcome assessments. On the other hand, some relevant strengthens deserve to be mentioned: the large sample size, the thorough assessment of ovarian reserve, the adjustment for possible confounders and the homogeneity in both medical and biological treatment thanks to the constant sharing and review of internal protocols. The achieved findings have the potential to improve counseling practices in the field of ART, enabling patients to gain a more comprehensive perspective on their infertility prognosis and the factors affecting IVF outcomes.

## Data Availability

The original contributions presented in the study are publicly available. This data can be found here: https://zenodo.org/records/13350396 10.5281/zenodo.13350395.

## References

[1] Yunakova M Slavov S Neykova C Shterev A Kostova I. Role of age as a major determinant of assisted reproductive technologies outcome. Proc Bulgarian Acad Sci. (2024) 77:753–9. doi: 10.7546/CRABS.2024.05.13

[2] Igarashi H Takahashi T Nagase S. Oocyte aging underlies female reproductive aging: biological mechanisms and therapeutic strategies. Reprod Med Biol. (2015) 14:159–69. doi: 10.1007/s12522-015-0209-5 PMC5715832 29259413

[3] Kwee J Elting ME Schats R McDonnell J Lambalk CB. Ovarian volume and antral follicle count for the prediction of low and hyper responders with *in vitro* fertilization. Reprod Biol Endocrinol. (2007) 5:9. doi: 10.1186/1477-7827-5-9 17362511 PMC1847823

[4] Moreau J Gatimel N Simon C Cohade C Lesourd F Parinaud J. Age-specific anti-Mullerian hormone (AMH) levels poorly affects cumulative live birth rate after intra-uterine insemination. Eur J Obstet Gynecol Reprod Biol X. (2019) 3:100043. doi: 10.1016/j.eurox.2019.100043 31403128 PMC6687367

[5] Xia X Burn MS Chen Y Karakaya C Kallen A. The relationship between H19 and parameters of ovarian reserve. Reprod Biol Endocrinol. (2020) 18:46. doi: 10.1186/s12958-020-00578-z 32404103 PMC7218823

[6] Broekmans FJ Soules MR Fauser BC. Ovarian aging: mechanisms and clinical consequences. Endocr Rev. (2009) 30:465–93. doi: 10.1210/er.2009-0006 19589949

[7] de Carvalho BR Rosa e Silva AC Rosa e Silva JC dos Reis RM Ferriani RA Silva de Sa MF. Ovarian reserve evaluation: state of the art. J Assist Reprod Genet. (2008) 25:311–22. doi: 10.1007/s10815-008-9241-2 PMC2596675 18679790

[8] Knauff EA Eijkemans MJ Lambalk CB ten Kate-Booij MJ Hoek A Beerendonk CC. Anti-Mullerian hormone, inhibin B, and antral follicle count in young women with ovarian failure. J Clin Endocrinol Metab. (2009) 94:786–92. doi: 10.1210/jc.2008-1818 19066296

[9] Anuradha K Partha Majumder S Shiffin R. Correlation of anti mullerian hormone and antral follicular count in ovarian reserve testing. Obstetrics Gynecol Res. (2022) 5(3):170–4. doi: 10.26502/ogr088

[10] Scheffer JB Carvalho RF Aguiar APS MaChado IJM Franca JB Lozano DM. Which ovarian reserve marker relates to embryo quality on day 3 and blastocyst; age, AFC, AMH? JBRA Assist Reprod. (2021) 25:109–14. doi: 10.5935/1518-0557.20200060 PMC7863095 32960526

[11] Practice Committee of the American Society for Reproductive Medicine. Electronic address aao, Practice Committee of the American Society for Reproductive M. Testing and interpreting measures of ovarian reserve: a committee opinion. Fertil Steril. (2020) 114:1151–7. doi: 10.1016/j.fertnstert.2020.09.134 33280722

[12] Kim HH. Markers of ovarian reserve: is it possible to estimate an ovarian age? Fertil Steril. (2017) 108:950–1. doi: 10.1016/j.fertnstert.2017.10.023 29202969

[13] Fouks Y Penzias A Neuhausser W Vaughan D Sakkas D. A diagnosis of diminished ovarian reserve does not impact embryo aneuploidy or live birth rates compared to patients with normal ovarian reserve. Fertil Steril. (2022) 118:504–12. doi: 10.1016/j.fertnstert.2022.06.008 35820943

[14] Katz-Jaffe MG Surrey ES Minjarez DA Gustofson RL Stevens JM Schoolcraft WB. Association of abnormal ovarian reserve parameters with a higher incidence of aneuploid blastocysts. Obstet Gynecol. (2013) 121:71–7. doi: 10.1097/AOG.0b013e318278eeda 23262930

[15] La Marca A Capuzzo M Longo M Imbrogno MG Spedicato GA Fiorentino F. The number and rate of euploid blastocysts in women undergoing IVF/ICSI cycles are strongly dependent on ovarian reserve and female age. Hum Reprod. (2022) 37:2392–401. doi: 10.1093/humrep/deac191 36006017

[16] Jaswa EG McCulloch CE Simbulan R Cedars MI Rosen MP. Diminished ovarian reserve is associated with reduced euploid rates via preimplantation genetic testing for aneuploidy independently from age: evidence for concomitant reduction in oocyte quality with quantity. Fertil Steril. (2021) 115:966–73. doi: 10.1016/j.fertnstert.2020.10.051 33583594

[17] Gat I AlKudmani B Wong K Zohni K Weizman NF Librach C. Significant correlation between anti-mullerian hormone and embryo euploidy in a subpopulation of infertile patients. Reprod BioMed. (2017) 35:602–8. doi: 10.1016/j.rbmo.2017.06.027 28826601

[18] Shim SH Ha HI Jung YW Shim SS Cho YK Kim JY. Maternal antimullerian hormone as a predictor of fetal aneuploidy occurring in an early pregnancy loss. Obstet Gynecol Sci. (2015) 58:494–500. doi: 10.5468/ogs.2015.58.6.494 26623414 PMC4663228

[19] Steiner AZ Pritchard D Stanczyk FZ Kesner JS Meadows JW Herring AH. Association between biomarkers of ovarian reserve and infertility among older women of reproductive age. JAMA. (2017) 318:1367–76. doi: 10.1001/jama.2017.14588 PMC5744252 29049585

[20] Busnelli A Somigliana E Cirillo F Levi-Setti PE. Is diminished ovarian reserve a risk factor for miscarriage? Results of a systematic review and meta-analysis. Hum Reprod. (2021) 27:973–88. doi: 10.1093/humupd/dmab018 34254138

[21] Committee EPCS Carvalho F Coonen E Goossens V Kokkali G Rubio C. ESHRE PGT Consortium good practice recommendations for the organisation of PGT. Hum Reprod Open. (2020) 2020:hoaa021. doi: 10.1093/hropen/hoaa021 32524036 PMC7257038

[22] Poseidon G Alviggi C Andersen CY Buehler K Conforti A De Placido G. A new more detailed stratification of low responders to ovarian stimulation: from a poor ovarian response to a low prognosis concept. Fertil Steril. (2016) 105:1452–3. doi: 10.1016/j.fertnstert.2016.02.005 26921622

[23] World Health Organization DoRHaR. WHO Laboratory Manual for the Examination and Processing of Human Semen. Geneva, Switzerland: World Health Organization. (2010).

[24] Ragni G Scarduelli C Calanna G Santi G Benaglia L Somigliana E. Blood loss during transvaginal oocyte retrieval. Gynecol Obstet Invest. (2009) 67:32–5. doi: 10.1159/000158649 18827490

[25] Cardellicchio L Reschini M Paffoni A Guarneri C Restelli L Somigliana E. Frozen-thawed blastocyst transfer in natural cycle: feasibility in everyday clinical practice. Arch Gynecol Obstet. (2017) 295:1509–14. doi: 10.1007/s00404-017-4383-z 28455581

[26] Cirillo F Immediata V Ronchetti C Carletti T Morenghi E Albani E. Steps forward in embryo transfer technique: a retrospective study comparing direct versus afterload catheters at different time frames. J Assist Reprod Genet. (2023) 40:2895–902. doi: 10.1007/s10815-023-02957-y PMC10656400 37819552

[27] Somigliana E Sarais V Reschini M Ferrari S Makieva S Cermisoni GC. Single oral dose of vitamin D(3) supplementation prior to *in vitro* fertilization and embryo transfer in normal weight women: the SUNDRO randomized controlled trial. Am J Obstet Gynecol. (2021) 225:283 e1– e10. doi: 10.1016/j.ajog.2021.04.234 33894153

[28] ESHRE. Ovarian stimulation for IVF/ICSI. In: Guideline of the European Society of Human Reproduction and Embryology. Grimbergen, Belgio: ESHRE (2019).

[29] Alpha Scientists in Reproductive Medicine and ESHRE Special Interest Group of Embryology. The Istanbul consensus workshop on embryo assessment: proceedings of an expert meeting. Hum Reprod. (2011) 26:1270–83. doi: 10.1093/humrep/der037 21502182

[30] Haviland MJ Murphy LA Modest AM Fox MP Wise LA Nillni YI. Comparison of pregnancy outcomes following preimplantation genetic testing for aneuploidy using a matched propensity score design. Hum Reprod. (2020) 35:2356–64. doi: 10.1093/humrep/deaa161 32856053

[31] Fan Y Chang Y Wei L Chen J Li J Goldsmith S. Apoptosis of mural granulosa cells is increased in women with diminished ovarian reserve. J Assist Reprod Genet. (2019) 36:1225–35. doi: 10.1007/s10815-019-01446-5 PMC6602993 30980221

[32] Aramesh S Alifarja T Jannesar R Ghaffari P Vanda R Bazarganipour F. Does vitamin D supplementation improve ovarian reserve in women with diminished ovarian reserve and vitamin D deficiency: a before-and-after intervention study. BMC Endocr Disord. (2021) 21:126. doi: 10.1186/s12902-021-00786-7 34154571 PMC8218405

[33] Ma R Song J Si J Liu Y Li X Cheng R. Acupuncture for diminished ovarian reserve: Protocol for a systematic review and meta-analysis. Med (Baltimore). (2019) 98:e16852. doi: 10.1097/MD.0000000000016852 PMC6716689 31441859

[34] Simpson S Pal L. Vitamin D and infertility. Curr Opin Obstet Gynecol. (2023) 35:300–5. doi: 10.1097/GCO.0000000000000887 37266579

[35] Tal R Seifer DB Wantman E Baker V Tal O. Antimullerian hormone as a predictor of live birth following assisted reproduction: an analysis of 85,062 fresh and thawed cycles from the Society for Assisted Reproductive Technology Clinic Outcome Reporting System database for 2012-2013. Fertil Steril. (2018) 109:258–65. doi: 10.1016/j.fertnstert.2017.10.021 29331235

[36] Barad DH Weghofer A Gleicher N. Utility of age-specific serum anti-Müllerian hormone concentrations. Reprod BioMed. (2011) 22:284–91. doi: 10.1016/j.rbmo.2010.12.002 21269880

[37] Erdogan K Sanlier N Utlu E Guvey H Kahyaoglu I Neselioglu S. Serum and follicular fluid thiol/disulfide homeostasis in diminished ovarian reserve patients undergoing *in vitro* fertilization/intracytoplasmic sperm injection treatment. Cureus. (2023) 15:e35476. doi: 10.7759/cureus.35476 36855584 PMC9968409

[38] Gutzeit O Bachar G Nebenzahl-Sharon K Weiner Z Beloosesky R Fainaru O. Hypoxia leads to diminished ovarian reserve in an age dependent manner. Res Square. (2024) 89(4):278–83. doi: 10.21203/rs.3.rs-2801535/v1 38569488

[39] Greene AD Patounakis G Segars JH. Genetic associations with diminished ovarian reserve: a systematic review of the literature. J Assisted Reprod Genet. (2014) 31:935–46. doi: 10.1007/s10815-014-0257-5 PMC4130940 24840722

[40] Jaillard S Bell K Akloul L Walton K McElreavy K Stocker WA. New insights into the genetic basis of premature ovarian insufficiency: Novel causative variants and candidate genes revealed by genomic sequencing. Maturitas. (2020) 141:9–19. doi: 10.1016/j.maturitas.2020.06.004 33036707

[41] Li N Xu W Liu H Zhou R Zou S Wang S. Whole exome sequencing reveals novel variants associated with diminished ovarian reserve in young women. Front Genet. (2023) 14:1154067. doi: 10.3389/fgene.2023.1154067 37065482 PMC10095150

[42] Sharma N Chakrabarti S. Ovarian Reserve. London, United Kingdom: Innovations In Assisted Reproduction Technology (2019).

[43] Barnabei A Strigari L Marchetti P Sini V De Vecchis L Corsello SM. Predicting ovarian activity in women affected by early breast cancer: A meta-analysis-based nomogram. Oncologist. (2015) 20:1111–8. doi: 10.1634/theoncologist.2015-0183 PMC4591948 26341758

[44] Hosseinzadeh F Kabodmehri R Mehrafza M Mansour-Ghanaei M Sorouri ZZ Gashti NG. The effect of age and AMH level on ART outcomes in patients with reduced ovarian reserve: A retrospective cross-sectional study. J Obstet Gynaecol India. (2022) 72:420–5. doi: 10.1007/s13224-021-01582-y PMC9568644 36458067

[45] te Velde ER Pearson PL. The variability of female reproductive ageing. Hum Reprod. (2002) 8:141–54. doi: 10.1093/humupd/8.2.141 12099629

[46] Steptoe PC Edwards RG. Birth after the reimplantation of a human embryo. Lancet. (1978) 2:366. doi: 10.1016/S0140-6736(78)92957-4 79723

[47] Alviggi C Humaidan P Howles CM Tredway D Hillier SG. Biological versus chronological ovarian age: implications for assisted reproductive technology. Reprod Biol Endocrinol. (2009) 7:101. doi: 10.1186/1477-7827-7-101 19772632 PMC2764709

[48] Wiweko B Prawesti DM Hestiantoro A Sumapraja K Natadisastra M Baziad A. Chronological age vs biological age: an age-related normogram for antral follicle count, FSH and anti-Mullerian hormone. J Assist Reprod Genet. (2013) 30:1563–7. doi: 10.1007/s10815-013-0083-1 PMC3843177 23955628

[49] Mahany EB Hershman DL Sauer MV Choi JM. Pregnancy despite ovarian insufficiency in a patient with breast cancer. Reprod Med Biol. (2013) 12:35–8. doi: 10.1007/s12522-012-0133-x PMC5904670 29699128

[50] Bi X Zhang J Cao D Sun H Feng F Wan X. Anti-Mullerian hormone levels in patients with gestational trophoblastic neoplasia treated with different chemotherapy regimens: a prospective cohort study. Oncotarget. (2017) 8:113920–7. doi: 10.18632/oncotarget.23027 PMC5768374 29371957

[51] Dolmans MM Hossay C Nguyen TYT Poirot C. Fertility preservation: how to preserve ovarian function in children, adolescents and adults. J Clin Med. (2021) 10. doi: 10.3390/jcm10225247 PMC8621487 34830528

[52] Zhang S Liu Q Chang M Pan Y Yahaya BH Liu Y. Chemotherapy impairs ovarian function through excessive ROS-induced ferroptosis. Cell Death Dis. (2023) 14:340. doi: 10.1038/s41419-023-05859-0 37225709 PMC10209065

[53] Bedoschi G Navarro PA Oktay K. Chemotherapy-induced damage to ovary: mechanisms and clinical impact. Future Oncol. (2016) 12:2333–44. doi: 10.2217/fon-2016-0176 PMC5066134 27402553

[54] Torella M Riemma G De Franciscis P La Verde M Colacurci N. Serum anti-mullerian hormone levels and risk of premature ovarian insufficiency in female childhood cancer survivors: systematic review and network meta-analysis. Cancers (Basel). (2021) 13. doi: 10.3390/cancers13246331 PMC8699404 34944951

[55] Sireesha MU Chitra T Subbaiah M Nandeesha H. Effect of laparoscopic ovarian cystectomy on ovarian reserve in benign ovarian cysts. J Hum Reprod Sci. (2021) 14:56–60. doi: 10.4103/jhrs.JHRS_94_20 34083993 PMC8057152

[56] Primarintan TN. The relationship between laparoscopic cystectomy and ovarian reserve: A systematic review and meta-analysis. Asian J Health Res. (2024) 3. doi: 10.55561/ajhr.v3i1.137

[57] La Marca A Sighinolfi G Radi D Argento C Baraldi E Artenisio AC. Anti-Mullerian hormone (AMH) as a predictive marker in assisted reproductive technology (ART). Hum Reprod. (2010) 16:113–30. doi: 10.1093/humupd/dmp036 19793843

[58] Ferraretti AP La Marca A Fauser BC Tarlatzis B Nargund G Gianaroli L. ESHRE consensus on the definition of 'poor response' to ovarian stimulation for *in vitro* fertilization: the Bologna criteria. Hum Reprod. (2011) 26:1616–24. doi: 10.1093/humrep/der092 21505041

[59] Cimadomo D Capalbo A Dovere L Tacconi L Soscia D Giancani A. Leave the past behind: women's reproductive history shows no association with blastocysts' euploidy and limited association with live birth rates after euploid embryo transfers. Hum Reprod. (2021) 36:929–40. doi: 10.1093/humrep/deab014 33608730

[60] Morin SJ Patounakis G Juneau CR Neal SA Scott RT Seli E. Diminished ovarian reserve and poor response to stimulation in patients <38 years old: a quantitative but not qualitative reduction in performance. Hum Reprod. (2018) 33:1489–98. doi: 10.1093/humrep/dey238 30010882

[61] Ebner T Sommergruber M Moser M Shebl O Schreier-Lechner E Tews G. Basal level of anti-Mullerian hormone is associated with oocyte quality in stimulated cycles. Hum Reprod. (2006) 21:2022–6. doi: 10.1093/humrep/del127 16679324

[62] Chiang T Schultz RM Lampson MA. Age-dependent susceptibility of chromosome cohesion to premature separase activation in mouse oocytes. Biol Reprod. (2011) 85:1279–83. doi: 10.1095/biolreprod.111.094094 PMC3223255 21865557

[63] Thomas MR Sparks AE Ryan GL Van Voorhis BJ. Clinical predictors of human blastocyst formation and pregnancy after extended embryo culture and transfer. Fertil Steril. (2010) 94:543–8. doi: 10.1016/j.fertnstert.2009.03.051 19409548

[64] Tarasconi B Tadros T Ayoubi JM Belloc S de Ziegler D Fanchin R. Serum antimüllerian hormone levels are independently related to miscarriage rates after *in vitro* fertilization-embryo transfer. Fertil Steril. (2017) 108:518–24. doi: 10.1016/j.fertnstert.2017.07.001 28865551

[65] Goldman KN Hodes-Wertz B McCulloh DH Flom JD Grifo JA. Association of body mass index with embryonic aneuploidy. Fertil Steril. (2015) 103:744–8. doi: 10.1016/j.fertnstert.2014.11.029 25576217

[66] Moini A Pirjani R Rabiei M Nurzadeh M Sepidarkish M Hosseini R. Can delivery mode influence future ovarian reserve? Anti-Mullerian hormone levels and antral follicle count following cesarean section: a prospective cohort study. J Ovarian Res. (2019) 12:83. doi: 10.1186/s13048-019-0551-z 31481111 PMC6720941

[67] Li HJ Seifer DB Tal R. AMH independently predicts aneuploidy but not live birth per transfer in IVF PGT-A cycles. Reprod Biol Endocrinol. (2023) 21:19. doi: 10.1186/s12958-023-01066-w 36739415 PMC9898926

[68] Kunicki M Skowrońska P Pastuszek E Jakiel G Smolarczyk R Łukaszuk K. Do serum androgens influence blastocysts ploidy in karyotypically normal women? Syst Biol Reprod Med. (2019) 65. doi: 10.1080/19396368.2019.1601295 30994373

[69] Arnanz A Bayram A Elkhatib I Abdala A El-Damen A Patel R. Antimullerian hormone (AMH) and age as predictors of preimplantation genetic testing for aneuploidies (PGT-A) cycle outcomes and blastocyst quality on day 5 in women undergoing *in vitro* fertilization (IVF). J Assist Reprod Genet. (2023) 40:1467–77. doi: 10.1007/s10815-023-02805-z PMC10310637 37145374

